# Unraveling the Anthocyanin Regulatory Mechanisms of White Mutation in *Verbena stricta* by Integrative Transcriptome and Metabolome Analysis

**DOI:** 10.3390/genes15121496

**Published:** 2024-11-21

**Authors:** Shengyue Chai, Jiaming Yang, Xiaofei Zhang, Xuwen Shang, Lixin Lang

**Affiliations:** Institute of Flowers, Liaoning Academy of Agricultural Sciences, Shenyang 110161, China; chaishengyue@163.com (S.C.); ycl60@163.com (J.Y.); zhxf_fei@126.com (X.Z.); 13998832008@163.com (X.S.)

**Keywords:** *Verbena stricta*, mutation, transcriptome, pigmentation, anthocyanins

## Abstract

**Background**: *Verbena stricta* is a perennial herb of the Verbenaceae family, known for its medicinal properties, wide adaptability, and high resistance. **Methods**: This research investigated the metabolic pathways of flower color change by combining transcriptome and metabolomics analyses. **Results**: In purple flowers and white variants, a total of 118 differentially accumulated metabolites (DAMs), including 20 anthocyanins, and 7627 differentially expressed genes (DEGs) were found. The downregulation of delphinidin-3-O-galactoside, delphinidin-3-O-glucoside, and delphinidin-3-O-(6″-O-p-coumaroyl) glucoside, along with the absence of petunidin and malvidin derivatives, may explain the loss of pigmentation in the white-flower mutant. Fourteen candidate genes involved in anthocyanin biosynthesis were identified, among which the expression of Flavonoid 3′, 5′-hydroxylase (F3′5′H) was significantly downregulated, notably limiting flux through the delphinidin pathway and reducing delphinidin accumulation. This limitation in upstream reactions, coupled with the multi-shunt process in downstream reactions, completely blocked the production of petunidin and malvidin. **Conclusions**: These findings offer new opinions on the anthocyanin metabolites and key genes responsible for the floral pigmentation in *V. stricta*. Additionally, the white variant provides a valuable platform for future research into the ornamental flower color of the Verbenaceae family.

## 1. Introduction

*V. stricta* has a graceful appearance and vibrant colors; this species, when in full bloom, resembles a captivating sea of purple flowers. Like *Aloysia triphylla* [1] and *Cordia verbenacea* [2], *V. stricta* holds medicinal value, particularly its heat-clearing and blood-cooling properties. Exhibiting robust growth and remarkable resistance to adverse conditions, this plant demands minimal management. In Northeast China, the lowest temperature in winter is approximately −30 °C, yet this plant can overwinter in the open field, resuming normal growth the following year. This perennial species has significant ornamental and research value, making it a noteworthy subject for further study.

Petal color is a significant phenotypic characteristic in plants because of its ease of observation and genetic stability, making it a valuable reference indicator for breeding work. Petal color is closely associated with pigment content, type, and distribution [3]. The primary chemical substances responsible for plant color in nature include flavonoids (such as anthocyanins), carotenoids, and betalains [4,5,6]. Most plants’ flowers, leaves, and fruit colors are predominantly caused by anthocyanins [7,8,9]. Anthocyanins possess a basic carbon skeleton of C6 (A-ring), C3 (C-ring), and C6 (B-ring). The different types of anthocyanins are formed due to methylation and hydroxylation modifications at various positions on the rings, primarily at the R1 and R2 positions on the B-ring. The three most common types of anthocyanins found in nature are cyanidin (Cy), pelargonidin (Pg), and delphinidin (Del) [3]. Cyanidin can be further modified to form peonidin (Pn), while delphinidin can be modified to form petunidin (Pt) and malvidin (Mv) [10].

The anthocyanin pathway typically uses phenylalanine as a precursor. This pathway consists of three stages, from phenylalanine to anthocyanins. The first stage starts with phenylalanine and ends with 4-coumaroyl CoA. The second phase is of paramount importance and leads to the synthesis of dihydroflavonol catalyzed by enzymes such as chalcone synthase (*CHS*) and chalcone isomerase (*CHI*). This is followed by the third phase, which is dedicated to the production of various anthocyanin compounds [11,12,13,14,15]. The enzymes and transcription factors play critical roles in regulating flower color formation by performing their respective functions [16,17]. The MBW complex is the main regulatory factor in anthocyanin synthesis, which is composed of MYB, bHLH, and WD40 [18,19,20]. Additionally, single transcription factors can regulate key synthase genes. The first discovery of a MYB family transcription factor in plants is *ZmC1*, which regulates anthocyanin expression in maize [21].

Recently, transcriptomic and metabolomic analyses have extensively studied the biosynthetic pathways associated with flavonoid metabolites in various plant species [22,23], including tomatoes, pears, strawberries, and grapes [24,25,26,27]. Multi-omic approaches enable more precise identification of genes responsible for synthesizing and regulating anthocyanins [28,29]. During a field survey of a resource garden, our team discovered a stable, inherited white-flower mutant of *V. stricta*. Therefore, the natural mutation in purple *V. stricta* flower (the white color of *V. stricta*) typically provides an excellent opportunity for in-depth studies of the complex metabolic networks and gene functions involved in anthocyanin synthesis.

This study integrates transcriptome and metabolomics technologies to infer the metabolic pathways associated with flower color changes in *V. stricta* and to examine candidate genes that may influence the loss of purple pigmentation. We aim to elucidate the molecular network governing flower color regulation, which has significant implications for understanding the color mechanisms in *V. stricta* and other ornamental flowers.

## 2. Materials and Methods

### 2.1. Plant Materials

Purple *V. stricta* Vent. (P) and white *V. stricta* Vent. (W) were grown at the Liaoning Academy of Agricultural Sciences, located in Shenyang City, China (41°48′11.75″ N, 123°25′31.18″ E). W is a natural mutation we found in the field; we had never seen white color before in *V. stricta*. In mid-to-late July, petal samples were taken at the full-blossom period (avoid taking tissues other than petals), thoroughly mixed, and subsequently stored at −80 °C. The petals were stored in the Key Laboratory of Floriculture Liaoning Provincial Liaoning Province for metabolome, transcriptome, and qRT-PCR analysis.

### 2.2. Metabolite Extraction and UPLC-ESI-MS/MS Conditions

The petals of two colors were vacuum freeze-dried and then pulverized with a mixer mill. The portion of the pulverized sample was placed in methanol solution and mixed with a vortex machine six times. The next day, the samples were subjected to high-speed centrifugation at 12,000 rpm for 10 min. After discarding the supernatant, the extract was filtered using a 0.22 μm microporous membrane. The filtered material was then analyzed using UPLC-ESI-MS/MS (UPLC, SHIMADZU Nexera X2, https://www.shimadzu.com.cn/ (accessed on 20 September 2021); MS, Applied Biosystems 4500 Q TRAP, https://www.thermofisher.cn/cn/zh/home/brands/applied-biosystems.html (accessed on 20 September 2021)). The analytical conditions were as follows: UPLC system equipped with an Agilent SB-C18 column (1.8 µm, 2.1 mm × 100 mm). The mobile phase comprised solvent A (0.1% formic acid in pure water) and solvent B (0.1% formic acid in acetonitrile). Sample analysis was conducted using a gradient program with an injection volume of 4 μL. The effluent was alternately linked to an ESI-equipped triple quadrupole-linear ion trap (QTRAP)-MS system.

### 2.3. Metabolite Data Analysis and Selection of Differential Metabolites

Mass spectrometry data were processed and analyzed with Analyst software (version 1.6.3). Metabolite identification and quantification were performed using the local metabolism database (Metware Bio-technology Co., Ltd., Wuhan, China), employing multiple reaction monitoring modes for quantitative analysis [30]. To enhance data normality, metabolite data were subjected to log2 transformation and normalized prior to statistical analysis. Significantly differential metabolites were screened using a Fold Change of ≥2 or ≤0.5 and a variable importance in projection (VIP) score of ≥1. The Kyoto Encyclopedia of Genes and Genomes (KEGG) database (https://www.kegg.jp/ (accessed on 25 August 2021)) was utilized to annotate DAMs and integrate metabolic pathways.

### 2.4. RNA Extraction and RNA-Seq

Frozen petal samples stored at −80 °C were taken out, and total RNA was extracted using the RNAprep Pure Plant Kit (Tiangen Biotech, Beijing, China). The RNA quality and integrity were tested using Agilent 2100 Bioanalyzer (Agilent Technologies, Santa Clara, CA, USA). After the library quality inspection was qualified, it was sequenced on the Illumina HiSeq 4000 platform. For RNA-seq details, refer to previous experimental studies [31,32,33].

### 2.5. Transcriptome Data Analysis and Functional Annotation

Raw data were rigorously processed using fastp (version 0.19.3) to generate clean reads for subsequent analysis. This process included removing reads with adapters, discarding low-quality reads, and eliminating poly-N reads. After obtaining clean reads, we used Trinity for de novo assembly to obtain reference sequences. The criteria for DEG screening were set at |log2Fold Change| ≥ 1 and *p*-value < 0.05 [34]. Analysis with the Gene Ontology (GO) and KEGG pathway databases was used for functional annotation of DEGs [35], and enrichment analysis was performed using the R package “clusterProfiler” [36]. Transcription factor analysis was performed using iTAK (version 1.7a), which identifies transcription factors based on predefined family classifications and rules in its database, using hmmscan for sequence alignment.

### 2.6. Association Analysis Between Metabolome and Transcriptome

DEGs and DAMs with Pearson correlation coefficients of >0.8 or <−0.8 and *p*-values < 0.05 were selected for constructing correlation networks for each pathway. The correlation network was presented using Cytoscape software (version 3.8).

### 2.7. qRT-PCR

Six DEGs (ANS, CHS, F3′5′H, BZ1, 3AT, and WRKY) were targeted for quantitative real-time PCR to verify their expression patterns in two materials. The primers were designed using the Primer5 software and synthesized by Sangon Biotech (Shanghai, China). The sequences of the primers are provided in Table S1. Quantitative real-time PCR was performed using the ABI Prism 7500 Real-Time PCR System (7500 and 7500 Fast Real-Time PCR System Software, V2.0.1, Foster City, CA, USA). The qRT-PCR method was performed following the procedures detailed in an earlier report [37]. The reaction was repeated three times biologically and technically, and the GAPDH sequence was used as an endogenous control. For the analysis of relative expression levels, the 2^−ΔΔCT^ method was used [38].

## 3. Results

### 3.1. Flavonoid Metabolome Profiling in Flowers of V. stricta

**Figure 1:**
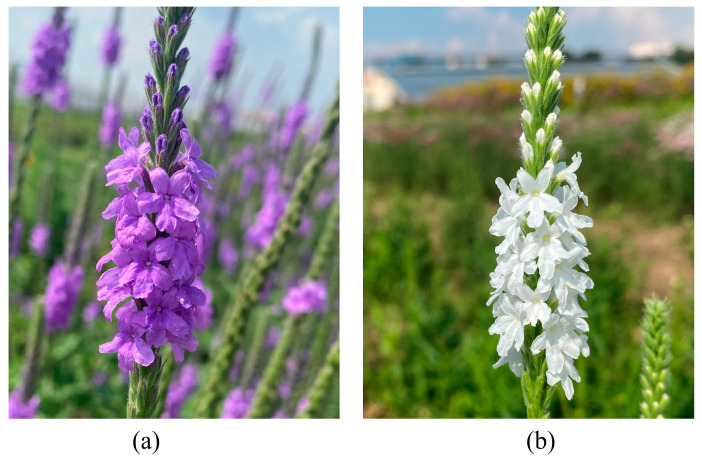
Phenotypes of (**a**) the purple *V. stricta* Vent. (P) and (**b**) white natural mutation (W).

**Figure 2:**
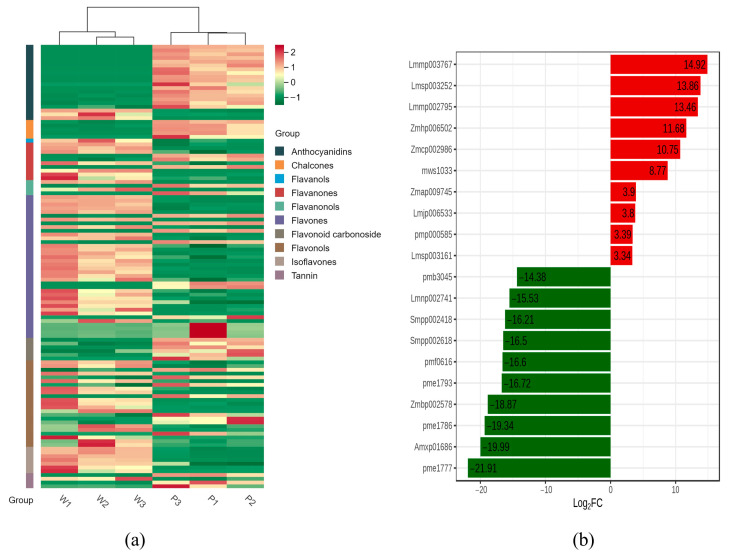
(**a**) Heat map of DAMs. (**b**) Top 20 DAMs. Different colors represent the values obtained after standardizing the relative metabolite content (red represents high content, green represents low content).

The fresh petals were collected from *V. stricta* with purple color (P) and its natural color mutant with white color (W) (Figure 1a,b). The UPLC-MS/MS system was used to analyze the metabolites of different color petal samples. The study of hierarchical clustering heat map results shows that three biological replicates have a close relationship, indicating high data reliability (Figure 2a).

Using the criteria of a Fold Change of ≥2 or ≤0.5, along with variable importance in projection of ≥1, the 118 differentially accumulated metabolites (DAMs) were classified into 10 categories, including Flavones (38), Tannin (4), Flavanonols (4), Flavanones (10), Chalcones (5), Flavonols (23), Anthocyanidins (20), flavonoid carbonoside (6), Flavones (1), and Isoflavones (7); in P and W petals, 63 metabolites content showed an increase, while 55 showed a decrease (Table S2, Figure 2a). As shown in Figure 2b, the highly significantly regulated DAMs were anthocyanidin compounds (pme1777, Amxp01686, pme1786, Zmbp002578, pme1793, pmf0616, Smpp002618, and Smpp002418), Flavonol compounds (Lmmp003767), and flavonoid carbonoside compounds (Lmnp002741).

A total of 20 anthocyanins were obtained; only Cyanidin-3-O-sophoroside-5-O-glucoside, Peonidin-3-O-rutinoside-5-O-glucoside, and Cyanidin-3-O-(6″-O-caffeoyl) glucoside were upregulated, while the remaining 17 anthocyanins were downregulated in white petals compared with purple petals. Interestingly, 12 anthocyanins had very high content in P petals but were basically not expressed in W petals (Table 1). This confirms that the reduction in anthocyanins is the main cause of white-flower mutations.

### 3.2. High-Throughput Transcriptome Sequencing and Analysis

Through transcriptome sequencing analysis, the key genes affecting the petal color mutations of P and W were identified. The six RNA-seq libraries were constructed, and a total of 58.81 Gb clean data was obtained. The percentages of Q30 and GC were between 93.61 and 94.21% and 44.36 and 44.86%, respectively, which indicates high-quality transcriptome data (Table S3).

The 77,121 unigene sequences obtained for predicting and analyzing the function of *V. stricta* were compared with seven databases, including GO and the KEGG, using BLASTX software to acquire gene annotation information. There were 34,317 (44.5%), 45,071 (58.44%), 33,915 (43.98%), 45,440 (58.92%), 30,770 (39.9%), 37,945 (49.2%), and 35,505 (46.04%) genes annotated in seven databases (Table S4). Through comparison with the NR database, it is possible to examine the similarity of transcript sequences of this species to those of closely related species, as well as the functional information of homologous sequences. In this study, Sesamum indicum and Handroanthus impetiginosus were found to be highly homologous to *V. stricta*, with 18,006 (39.95%) and 10,402 (23.08%) unigenes annotated, respectively (Figure S1).

**Figure 3:**
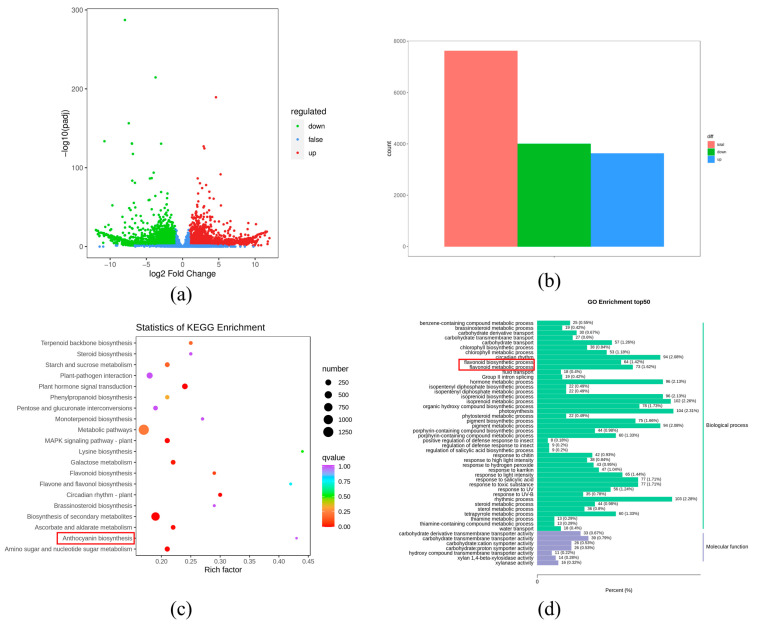
(**a**) Volcano figure of DEGs between P vs. W; (**b**) the number of the DEGs between P vs. W; (**c**) KEGG-enriched pathway analysis; (**d**) fifty GO enrichment pathway between white and purple flowers in *V. stricta*.

According to their expression levels in different samples, DEGs were identified. Using |log2Fold Change| >= 1 and FDR < 0.05, 7627 DEGs were identified in P vs. W, including 3628 upregulated genes and 3999 downregulated genes (Figure 3a,b).

### 3.3. Function Analysis of DEGs by KEGG and GO Database

To verify the biological functions of DEGs in P and W samples, we performed GO enrichment analysis and KEGG pathway enrichment. In total, 4829 genes were successfully annotated in the GO database, mainly classified into cellular components, biological processes, and molecular function. In biological processes, 64 DEGs (1.42%) were associated with the flavonoid biosynthetic process, and 73 DEGs (1.62%) were involved in the flavonoid metabolic process (Figure 3d, Table S5). There were 2919 DEGs mapped to 135 pathways of the KEGG database. Anthocyanin biosynthesis (ko00942) was one of the most significant enrichment pathways (Figure 3c, Table S6).

### 3.4. Transcription Factors (TFs) of DEG Analysis

**Figure 4:**
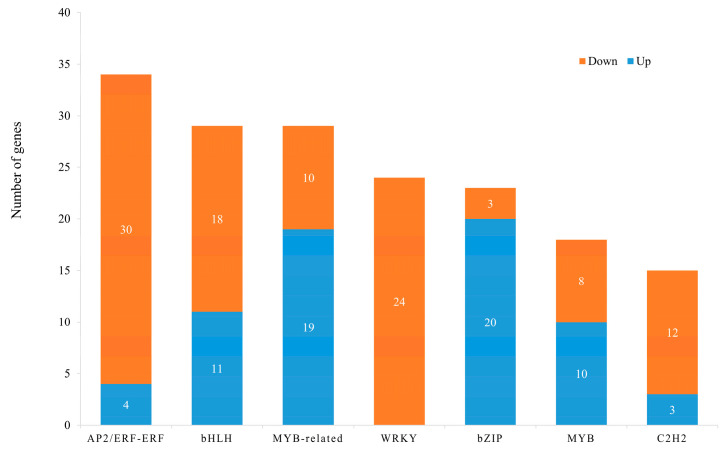
The number of differentially expressed transcription factors between white and purple flowers in *V. stricta*.

In total, 493 differential transcription factors were identified between white and purple petals, of which 289 were downregulated and 204 were upregulated. ERF (34), MYB (18), MYB-related (29), bZIP (23), BHLH (29), and C2H2 (15), which were related to anthocyanin biosynthesis, were found in this study (Figure 4). It is noteworthy that all 24 WRKY TFs were downregulated. The upregulated genes in the bZIP family were only three, and the downregulated genes of the ERF family had four. These TFs probably have crucial roles in regulating the anthocyanin biosynthetic processes involved in color change in *V. stricta*.

### 3.5. Association Analysis Between Metabolome and Transcriptome in Flavonoid Metabolism Pathway

**Figure 5:**
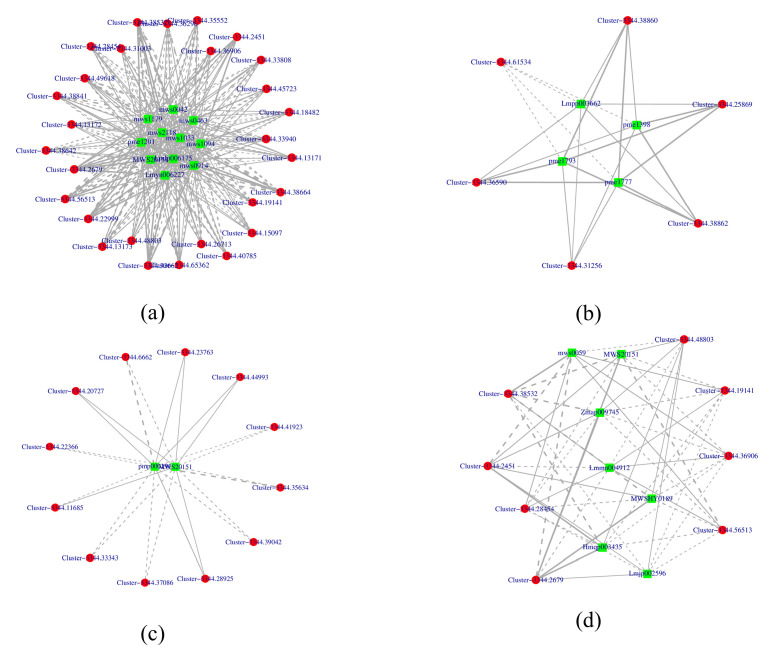
Association analysis between metabolome and transcriptome: (**a**) Ko00941; (**b**) Ko00942; (**c**) Ko00943; (**d**) Ko00944; flavonoid biosynthesis (ko00941), anthocyanin biosynthesis (ko00942), Isoflavonoid biosynthesis (ko00943), flavone and flavonol biosynthesis (ko00944); green: metabolite; red: gene; solid lines: positive correlation; dashed lines: negative correlation.

A Pearson correlation coefficient analysis (Pearson correlation coefficient of >0.8 or <−0.8, *p*-value < 0.05) was performed on the DEGs and DAMs to reveal the changes in white mutants. A total of 54 genes, shown in red circles, and 24 metabolites, shown in green squares, of four KEGG pathways (Ko00941, Ko00942, Ko00943, and Ko00944) were presented using Cytoscape software (version 3.8). Within these, Ko00942 was composed of 10 nodes and 24 edges, with 4 pairs correlating negative and 20 pairs correlating positive. The results, as shown in Figure 5, indicate a strong correlation between related genes and metabolites. The construction of transcript–metabolite correlation networks highlights the significant role of flavonoid metabolic pathways in the formation of flower color. Detailed information on genes and metabolites in each pathway can be found in the Supplementary Materials, Table S7.

### 3.6. Candidate Genes Responsible for the Absence of Purple in V. stricta

To obtain more gene information about the color formation of *V. stricta*, genes associated with petal pigmentation were analyzed from the transcriptome database. A total of 52 unigenes encoding 14 enzymes were analyzed on the basis of standard gene names and synonyms in KEGG functional annotations, which had 20 downregulated and only three upregulated unigenes in white petals compared with purple petals (Table 2, Table S8).

**Figure 6:**
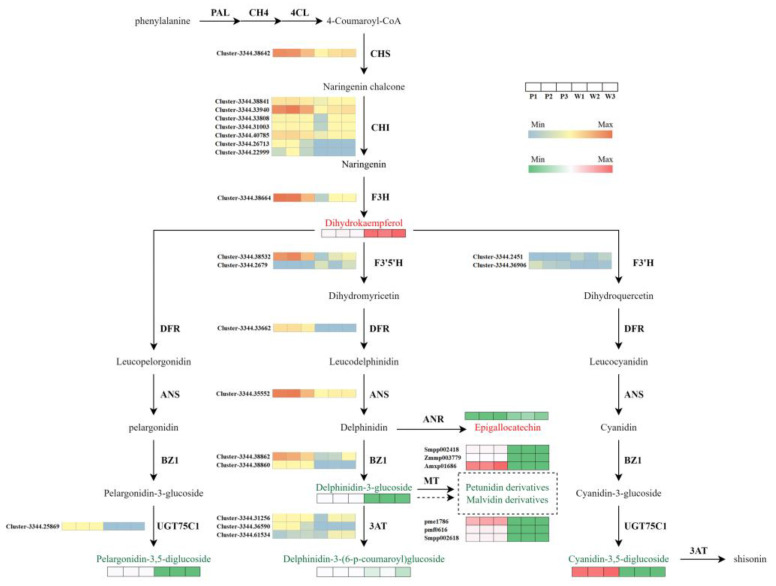
Biosynthetic pathway of anthocyanin in purple petals of P and white petals of W. Gene expression in the heatmaps was scaled using FPKM values; metabolite heatmaps were constructed based on the relative content of metabolites; red metabolites indicate upregulation, while green metabolites indicate downregulation.

Anthocyanins belong to the flavonoid family, and the pathway for anthocyanin biosynthesis is a crucial route that determines the color expression of plant organs. According to our findings, we reconstructed the anthocyanin biosynthesis pathway in *V. stricta*. As shown in Figure 6, key genes related to the anthocyanin biosynthesis pathway were analyzed, including early genes CHI, CHS, and F3H (flavanone-3-hydroxylase) and late genes F3′H (flavonoid 3′-monooxygenase), F3′5′H, DFR (dihydroflavonol 4-reductase), and ANS (anthocyanidin synthase), as well as genes related to the formation of anthocyanin derivatives in later stages: UGT75C1 (anthocyanidin 3-O-glucoside 5-O-glucosyltransferase), BZ1 (anthocyanidin 3-O-glucosyltransferase), and 3AT (anthocyanidin 3-O-glucoside 6″-O-acyltransferase). In the white mutant, most genes were significantly downregulated, which may be the primary reason for the absence of anthocyanin.

### 3.7. QRT-RCR Analysis of Gene Expression

**Figure 7:**
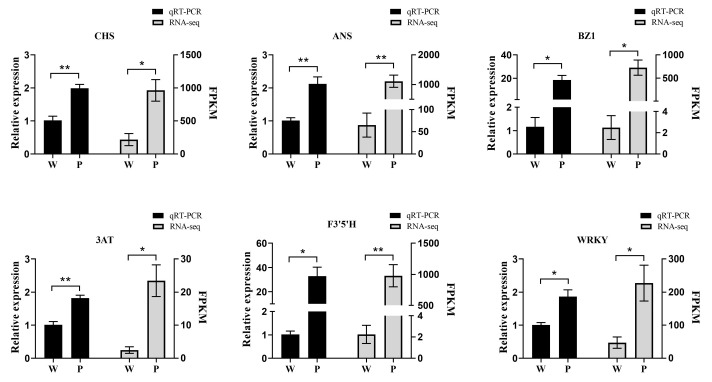
Verification of genes related to anthocyanin biosynthesis by qRT-PCR analysis. Asterisks denote t-test significance: * *p* < 0.05; ** *p* < 0.01.

To confirm the reliability of the transcriptome data, six genes (ANS, WRKY, CHS, BZ1, F3′5′H, and 3AT) related to anthocyanin metabolism were chosen for validation of the sequencing results. The qRT-PCR results indicated that the six genes expressing trends were nearly consistent with the transcript abundance patterns (Figure 7); Table S1 shows the primer sequences of 6 genes.

## 4. Discussion

### 4.1. Flavonoid Metabolite Differences Are Responsible for the Color Change of Two Materials

In the last few years, color mutants have been extensively used in plant color studies, particularly in ornamental plants [39,40]. Color formation in nature results from the combined action of multiple factors, including both internal and external elements, such as petal tissue structure, temperature, light, environmental pH, mineral nutrients, and plant hormones. However, anthocyanins are the most significant factor influencing flower color [12]. Anthocyanins are an extensively studied pigment group in plants, providing the foundational basis for the coloration of most plants in nature [40]. Our study identified 20 differentially expressed anthocyanins, 17 of which were downregulated in white flowers, with 12 being nearly absent (Table 1), indicating that the blockage of anthocyanin biosynthesis is the crucial cause of the loss of color in white flowers.

Malvidin, petunidin, and delphinidin are the main anthocyanins that impart blue-purple hues to plants and have been confirmed in several species, including grape hyacinth, potato, and grapevine [41,42,43]. Our metabolome analysis showed that petunidin and malvidin derivatives were highly expressed in purple flowers but were nearly undetectable in white flowers; moreover, upstream delphinidins showed a marked downregulation (Table 1). So, the downregulation of delphinidin-3-O-galactoside, delphinidin-3-O-glucoside, and delphinidin-3-O-(6″-O-p-coumaroyl) glucoside, coupled with the absence of petunidin and malvidin derivatives, are responsible for the color absence in the white-flower mutant. Additionally, delphinidin-3-O-glucoside and delphinidin-3-O-(6″-O-p-coumaroyl) glucoside were identified as key metabolite nodes in the correlation network of DEGs and DAMs (Figure 5). This observation is consistent with earlier studies that identified a lack of malvidin and petunidin compounds in cream-colored alfalfa flowers [44].

### 4.2. Genes Involved in the Anthocyanin Pathway Influence the Flower Color in Two Materials

Transcriptome analysis identified 7627 DEGs in total. Further KEGG enrichment analysis revealed that these DEGs are predominantly enriched in the biosynthesis pathways of flavonoids and anthocyanins (Figure 3). Most structural genes, including CHS, F3′5′H, ANS, BZ1, and 3AT, involved in flavonoid synthesis showed significantly higher transcription expression in purple petals than in white petals (Table 2). This observation suggests a positive correlation between anthocyanin accumulation and the expression of genes encoding enzymes, consistent with previous research findings [45,46,47,48,49,50].

MYB and bHLH are critical transcription factors in anthocyanin synthesis [51]. Consistent with previously reported studies, a retrotransposon insertion mutation in VvMYBA1 is the primary factor preventing anthocyanin synthesis in white grape varieties [52]. Additionally, light exposure can affect the stability of MdMYB1, thereby increasing anthocyanin accumulation in apples [53]. In addition, MYB and bHLH often collaborate to regulate anthocyanin transcription [54]. For example, in maize, C1 (MYB) interacts with R (bHLH) to recognize the CAACACC element, thereby regulating the tissue-specific expression of anthocyanins [55]. Xie et al. found that MdbHLH3, an important gene regulating anthocyanin expression, binds to the promoters of MdMYB10 and MdDFR, a finding consistent with similar research into nectarines [56,57]. In our study, differentially expressed transcription factors MYB (18) and BHLH (29) were identified in two materials, which may act as candidates to regulate anthocyanin biosynthesis in *V. stricta* (Figure 4).

### 4.3. Reason for the Loss of Purple Color in V. stricta Through the Delphinidin Synthesis Pathway

Our metabolism result has revealed that the color variation between purple and white flowers in *V. stricta* is caused by the lower expression of delphinidin and the loss of petunidin and malvidin. Therefore, we focus on the Del synthesis pathway to identify key genes related to the lack of purple pigmentation. In this pathway, 1CHS (Cluster-3344.38642), 7CHI (Cluster-3344.38841; Cluster-3344.33940; Cluster-3344.33808; Cluster-3344.31003; Cluster-3344.40785; Cluster-3344.26713; Cluster-3344.22999), 1F3H (Cluster-3344.38664), 1F3′5′H (Cluster-3344.38532), 1DFR (Cluster-3344.33662), and 1ANS (Cluster-3344.35552) showed lower transcript abundance in white petals compared with the purple petals (Table 2, Figure 6). The analysis and comparison of these candidate genes are important for finding key genes for the absence of flower anthocyanins. The first step in anthocyanin synthesis is catalyzed by CHS [58]. Subsequently, naringenin chalcone is converted into dihydrokaempferol, a substrate essential for anthocyanin synthesis, through the catalytic actions of CHI and F3H [59]. Interestingly, in our study, white petals had a higher accumulation of dihydrokaempferol, suggesting that the blockage of flower color formation occurs downstream in the pathway.

F3′5′H is a crucial gene for the synthesis of blue-purple anthocyanins, directing the pathway toward the formation of trihydroxylated delphinidin by controlling the position and number of B-ring hydroxylation [60]. Previous studies have demonstrated that co-transformation of F3′5′H and DFR in *Torenia fournieri* successfully obtained new blue-purple carnations and roses [61,62]. Similarly, Brugliera et al. reported that F3′5′H promoted the accumulation of delphinidin in transgenic chrysanthemum plants [63]. In our study, the transcripts of F3′5′H sequences in white flowers showed significantly lower levels of gene transcript, more than 1000-fold (Figure 7). This dramatic reduction in F3′5′H expression inhibited anthocyanin formation, leading to decreased anthocyanin accumulation. Furthermore, we deduce that the lower expression of F3′5′H could reduce the production of dihydromyricetin compounds, which may subsequently inhibit the production of DFR and consequent ANS.

Numerous studies have shown that loss-of-color adaptations can be performed in various ways. For instance, the mutation of the single early anthocyanin synthesis gene *CHS* can turn purple violets into white flowers [64]. In soybeans, the flower color is regulated by the recessive allele of the w4 locus of the late gene *DFR2*, with the loss of *DFR2* function leading to a completely white phenotype [65]. Regulating branch points in anthocyanin synthesis is a novel approach to exploring color-deficient phenotypes. For instance, transient interference with the expression of the fcmyb1 in white strawberries fruit inhibited the expression of ANR (Anthocyanidin reductase) and LAR (Leucounthcyanidin4-reductase), thereby increasing anthocyanin content [66]. Changes in substrate competition between FLS (flavonol synthesis) and DFR in grape hyacinths may also lead to the loss of blue pigments [42]. We also discussed the impact of other flavonoid metabolic route branches on purple pigmentation. Delphinidin is a branch point in late-stage anthocyanin biosynthesis, being intermediate in the production of both the colored delphinidin-3-glucoside catalyzed by BZ1 and the colorless epigallocatechin catalyzed by ANR. Due to the competition for similar substrates, the accumulation in epigallocatechin might be closely associated with the decrease in delphinidin-3-glucoside. Our results showed that the white petals of *V. stricta* had a 2.29-fold higher epigallocatechin content than purple flowers (Figure 6). The competition with epigallocatechin likely further reduces the contents of colored anthocyanin derivatives. Delphinidin-3-glucoside, which is the substrate for the biosynthesis of petunidin and malvidin, is inherently limited through its previous biosynthesis process. Moreover, the competitive biosynthesis of the colorless epigallocatechin exacerbates this limitation, thereby significantly restricting the synthesis of malvidin and petunidin pigments. Additionally, the suppressed expression of the *3AT* gene inhibits the synthesis of delphinidin-3-O-(6″-O-p-coumaroyl) glucoside. In conclusion, F3′5′H is identified as the most likely target responsible for the absence of purple color in *V. stricta*. Its low expression effectively limits the flux in the Del pathway. It reduces the accumulation of Del. The restriction of upstream reactions, combined with the multi-shunt process of downstream reactions, completely block the generation of petunidin and malvidin. The disappearance of stable-colored compounds leads to the loss of colored phenotypes in *V. stricta*.

## Figures and Tables

**Table 1:** Differentially accumulated anthocyanins in P and W.

Metabolite	Content P	Content W	VIP	Fold Change	Type
Cyanidin-3-O-sophoroside-5-O-glucoside	9.00 × 10^0^	1.54 × 10^4^	1.27	1.72 × 10^3^	Up
Peonidin-3-O-rutinoside-5-O-glucoside	3.04 × 10^3^	1.65 × 10^4^	1.22	5.45 × 10^0^	Up
Cyanidin-3-O-(6″-O-caffeoyl) glucoside	2.19 × 10^5^	9.82 × 10^5^	1.26	4.49 × 10^0^	Up
Delphinidin-3-O-(6″-O-p-coumaroyl) glucoside	1.63 × 10^5^	6.11 × 10^4^	1.19	3.75 × 10^−1^	Down
Cyanidin-3-O-glucoside-5-O-diglucuronide	1.32 × 10^5^	4.42 × 10^4^	1.25	3.36 × 10^−1^	Down
Pelargonidin-3,5,7′-tri-O-glucuronide	9.24 × 10^6^	1.11 × 10^6^	1.27	1.20 × 10^−1^	Down
Delphinidin-3-O-galactoside	1.03 × 10^5^	4.83 × 10^3^	1.25	4.67 × 10^−2^	Down
Delphinidin-3-O-glucoside	9.71 × 10^4^	2.91 × 10^3^	1.25	2.99 × 10^−2^	Down
Petunidin-3-O-(6″-O-p-coumaroyl) rutinoside	4.12 × 10^4^	9.00 × 10^0^	1.27	2.19 × 10^−4^	Down
Delphinidin-3-O-(2‴-O-p-coumaroyl) rutinoside	4.77 × 10^4^	9.00 × 10^0^	1.27	1.89 × 10^−4^	Down
Cyanidin-3-O-(2″-O-xylosyl) galactoside	6.23 × 10^4^	9.00 × 10^0^	1.27	1.44 × 10^−4^	Down
Peonidin-3,5-O-diglucoside	1.34 × 10^5^	9.00 × 10^0^	1.27	6.74 × 10^−5^	Down
Petunidin-3-O-(6″-O-Acetyl) glucoside	6.80 × 10^5^	9.00 × 10^0^	1.28	1.32 × 10^−5^	Down
Malvidin-3-O-(6″-O-acetyl) glucoside	8.32 × 10^5^	9.00 × 10^0^	1.27	1.08 × 10^−5^	Down
Malvidin-3-O-(6″-O-acetyl) glucoside-5-O-glucoside	8.93 × 10^5^	9.00 × 10^0^	1.27	1.01 × 10^−5^	Down
Pelargonidin-3,5-O-diglucoside	9.72 × 10^5^	9.00 × 10^0^	1.27	9.26 × 10^−6^	Down
Cyanidin-3-O-(6″-O-acetyl) glucoside-5-O-glucoside	4.30 × 10^6^	9.00 × 10^0^	1.27	2.09 × 10^−6^	Down
Malvin	5.99 × 10^6^	9.00 × 10^0^	1.27	1.50 × 10^−6^	Down
Petunidin-3,5-di-O-glucoside	9.37 × 10^6^	9.00 × 10^0^	1.27	9.61 × 10^−7^	Down
Cyanin	3.55 × 10^7^	9.00 × 10^0^	1.27	2.53 × 10^−7^	Down

P, purple *V. stricta*; W, white *V. stricta*; content: the average of three replicates of the relative content of the P and W petal samples; differentially accumulated compounds were identified using the following criteria: variable importance in projection (VIP) ≥ 1, Fold Change ≥2 or ≤0.5; type represents the expression trend of white flowers compared with purple flowers.

**Table 2:** Candidate genes involved in color changes of *V. stricta*.

Function	Gene	Enzyme	KO id (EC-No.)	^1^ All	^2^ Up	^3^ Down
Anthocyanin biosynthesis	*BZ1*	Anthocyanidin 3-O-glucosyltransferase	K12930 (2.4.1.115)	2	0	2
	*3AT*	Anthocyanidin 3-O-glucoside 6″-O-acyltransferase	K21383 (2.3.1.215)	9	1	2
	*UGT75C1*	Anthocyanidin 3-O-glucoside 5-O-glucosyltransferase	K12338 (2.4.1.298)	1	0	1
	*F3′5′H*	Flavonoid 3′,5′-hydroxylase	K13083 (1.14.14.81)	3	1	1
	*F3′H*	Flavonoid 3′-monooxygenase	K05280 (1.14.14.82)	2	1	1
Flavonoid biosynthesis	*CHS*	Chalcone synthase	K00660 (2.3.1.74)	1	0	1
	*F3H*	Flavanone 3-dioxygenase	K00475 (1.14.11.9)	1	0	1
	*DFR*	Dihydroflavonol 4-reductase	K13082 (1.1.1.219)	2	0	1
	*CYP98A*	5-O-(4-coumaroyl)-D-quinate 3′-monooxygenase	K09754 (1.14.14.96)	3	0	1
	*ANS*	Anthocyanidin synthase	K05277 (1.14.20.4)	2	0	1
	*FLS*	Flavonol synthase	K05278 (1.14.20.6)	7	0	1
	*CYP73A*	Trans-cinnamate 4-monooxygenase	K00487 (1.14.14.91)	4	0	0
	*ANR*	Anthocyanidin reductase	K08695 (1.3.1.77)	1	0	0
	*CHI*	Chalcone isomerase	K01859 (5.5.1.6)	14	0	7

^1^ All: the total number of unigenes; ^2^ Up: the number of significantly upregulated genes in P and W; ^3^ Down: the number of significantly downregulated genes in P and W.

## Data Availability

The RNA-seq data have been submitted to NCBI SRA: PRJNA1167914.

## Supplementary Materials

The following supporting information can be downloaded at: https://www.mdpi.com/article/10.3390/genes15121496/s1, Figure S1: unigene species. stat, Table S1: primer sequences of genes used for qPCR verification, Table S2: types and contents of differently expressed flavonoid compounds in P and W, Table S3: the sequences information by RNA-seq, Table S4: all unigene annotation, Table S5: the Go terms enriched by the DEGs, Table S6: the KEGG-enriched pathway by DEGs, Table S7: gene–metabolite pairs involved in four KEGG pathway, Table S8: differentially expressed genes in this study.
