# Supplementary figures and images for: Unraveling the Anthocyanin Regulatory Mechanisms of White Mutation in Verbena stricta by Integrative Transcriptome and Metabolome Analysis
Source: Genes (Basel). 2024 Nov 21;15(12):1496. doi: 10.3390/genes15121496 (PMC11675223; doi:10.3390/genes15121496)

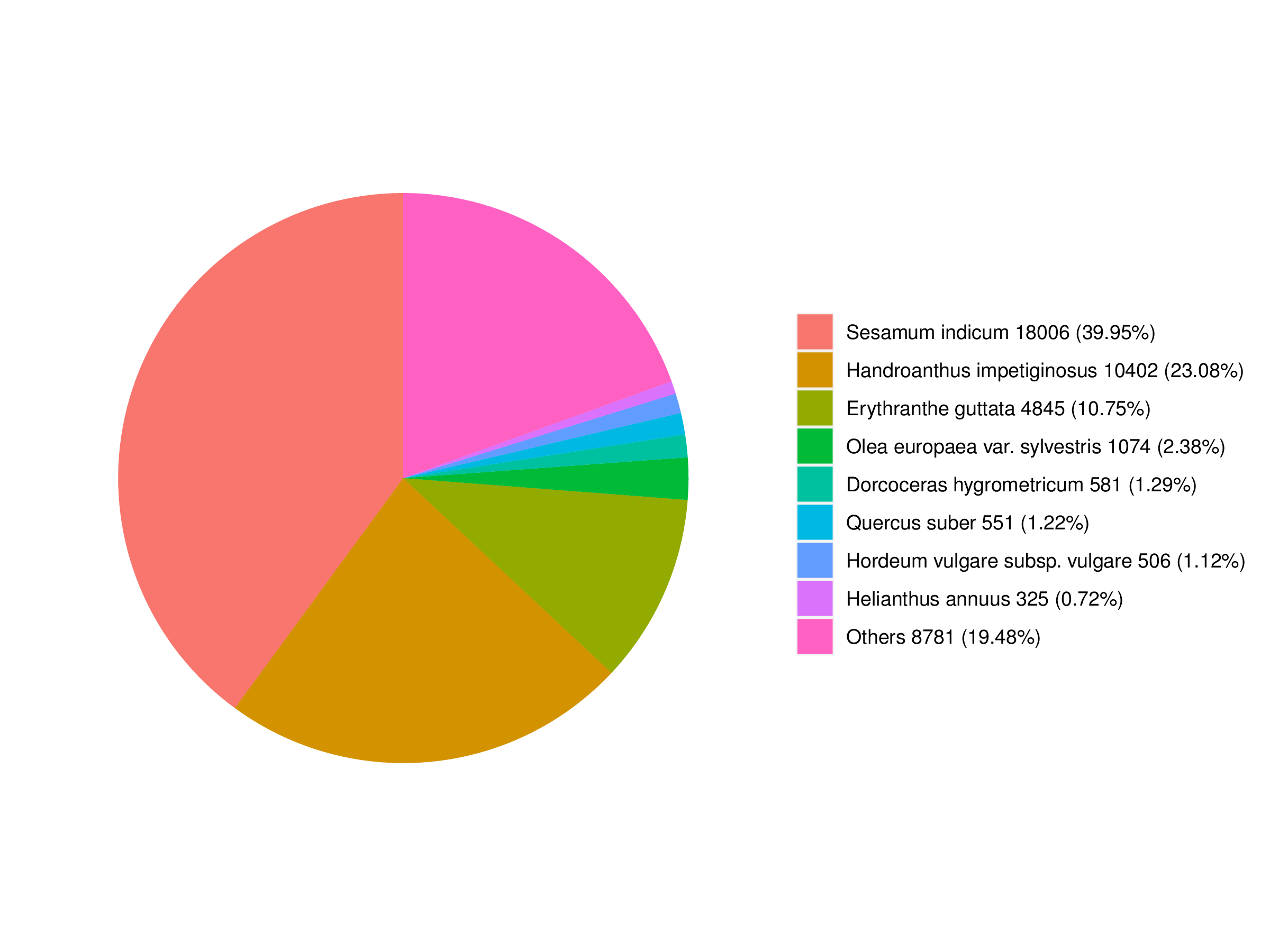

Supplement: Supplementary file 1 [file genes-15-01496-s001.zip › supplementary files/supplementary figure/Figuer S1 Unigene species.stat.png]
